# Optimizing Strategies in Patients Affected by Tumors Infiltrating the Skull: A Single Center Experience

**DOI:** 10.3390/brainsci15040420

**Published:** 2025-04-20

**Authors:** Giuseppe Emmanuele Umana, Sruthi Ranganathan, Salvatore Marrone, Jessica Naimo, Matteo Giunta, Angelo Spitaleri, Marco Fricia, Gianluca Ferini, Gianluca Scalia

**Affiliations:** 1 Department of Neurosurgery, Trauma and Gamma Knife Center, Cannizzaro Hospital, 95126 Catania, Italy; umana.nch@gmail.com (G.E.U.); angelospitaleri@inwind.it (A.S.); marco.fricia@aoec.it (M.F.); 2 School of Medicine and Surgery, Kore University of Enna, 94100 Enna, Italy; gianluca.ferini@grupposamed.com; 3 Department of Medicine, University of Cambridge, Cambridge CB2 0QQ, UK; sr932@cam.ac.uk; 4 Department of Neurosurgery, Sant’ Elia Hospital, 93100 Caltanissetta, Italy; salvo.mr89@gmail.com; 5 Pain Therapy and Palliative Care Unit, ASP 7 Ragusa, 97100 Ragusa, Italy; jessica.naimo@asp.rg.it; 6 MT Ortho srl, 95025 Aci Sant’Antonio, Italy; matteo.giunta@mtortho.com; 7 Department of Radiation Oncology, REM Radioterapia srl, 95029 Viagrande, Italy; 8 Neurosurgery Unit, Department of Head and Neck Surgery, Garibaldi Hospital, 95124 Catania, Italy

**Keywords:** mixed reality, cranioplasty, tumor removal, skull reconstruction, neuro-oncology, intraoperative navigation, custom-made titanium mesh

## Abstract

**Background:** One-step cranioplasty combined with tumor removal is a recognized approach in neuro-oncology for patients with neoplastic skull invasion. The use of advanced technologies, including Mixed Reality (MR), has introduced new possibilities in surgical workflows. MR technology may provide additional benefits in preoperative planning, patient engagement, and intraoperative guidance. Can the proposed treatment algorithm, which includes Mixed Reality (MR) for preoperative planning and intraoperative navigation, demonstrate tangible utility and improve outcomes in the surgical management of skull-invasive tumors? **Methods:** A retrospective study was conducted on 14 patients treated at Cannizzaro Hospital, Catania, Italy, for skull-invasive tumors. The treatment algorithm incorporated tumor removal and one-step cranioplasty using custom-made titanium alloy meshes. Standard intraoperative navigation was compared with MR-based navigation. MR headsets and the Virtual Surgery Intelligence (VSI) platform were employed for preoperative planning, surgical guidance, and patient/family communication. Tumor types included nine meningiomas and five other tumor variants. **Results:** The integration of MR proved beneficial for preoperative planning, facilitating enhanced visualization of patient anatomy and aiding communication with patients and families. MR-assisted intraoperative navigation offered improved anatomical familiarity but demonstrated slightly lower accuracy compared with standard navigation. Postoperative outcomes were satisfactory across the cohort, with no significant complications reported. **Conclusions:** The study highlights the potential utility of the proposed treatment algorithm including MR technology in the surgical management of skull-invasive tumors. While MR provides enhanced visualization and preoperative engagement, standard navigation remains more precise during surgery. Nevertheless, MR serves as a valuable complementary tool, and its role in neuro-oncological workflows is expected to grow with technological advancements.

## 1. Introduction

In neuro-oncology, one-step cranioplasty combined with tumor removal has become a well-established strategy allowing for optimal skull reconstruction in cases where the tumor invades the skull [1,2].

In this context, “one-step cranioplasty” refers to a surgical strategy in which tumor resection and cranial reconstruction with a custom-made prosthesis are performed during the same operative session. This approach enables immediate anatomical restoration, aesthetic repair, and brain protection. Various biocompatible materials such as methyl methacrylate, hydroxyapatite, titanium alloy mesh, ceramics, and polyetheretherketone (PEEK) are utilized for skull reconstruction following bone removal [3,4]. Accurate preoperative and intraoperative planning is essential in this context. Intraoperative navigation systems, including optical or magnetic methods along with Mixed Reality (MR), provide valuable tools to enhance surgeon familiarity with the patient’s anatomy both before and during surgery, facilitating the simulation of the final cranioplasty position. The potential utility of MR extends even to the informed consent phase, aiding in the comprehensive explanation of surgical strategies, associated risks, anticipated cosmetic outcomes, and treatment goals to patients and their families [5]. This study aims to present our workflow for one-step cranioplasty in patients with skull-invading tumors. By sharing our experience with MR in cranioplasty planning and intraoperative navigation for oncologic patients, we explore the current role of MR in this patient population, highlighting the existing technical limitations and envisioning future applications.

The main objective of this study is to investigate whether integrating MR navigation into one-step cranioplasty for skull-invading tumors can provide concrete clinical benefits. We specifically focus on (i) the preoperative advantages of MR in surgical planning and patient counseling, (ii) its intraoperative accuracy and ease of use compared with standard optical navigation, and (iii) its impact on patient-centered outcomes such as satisfaction and understanding of the procedure.

While standard neuronavigation has become a staple in cranial procedures, recent advances in MR technology enable surgeons to overlay three-dimensional (3D) holographic patient-specific anatomical models onto the patient’s own anatomy. Unlike conventional navigation systems that require the surgeon to reference a separate monitor, MR allows for direct “heads-up” visualization through a head-mounted display. This immersive interaction with holographic reconstructions offers a fundamentally different, potentially more intuitive approach to preoperative planning and intraoperative guidance, distinguishing MR technology from existing solutions.

## 2. Material and Methods

**Figure 1:**
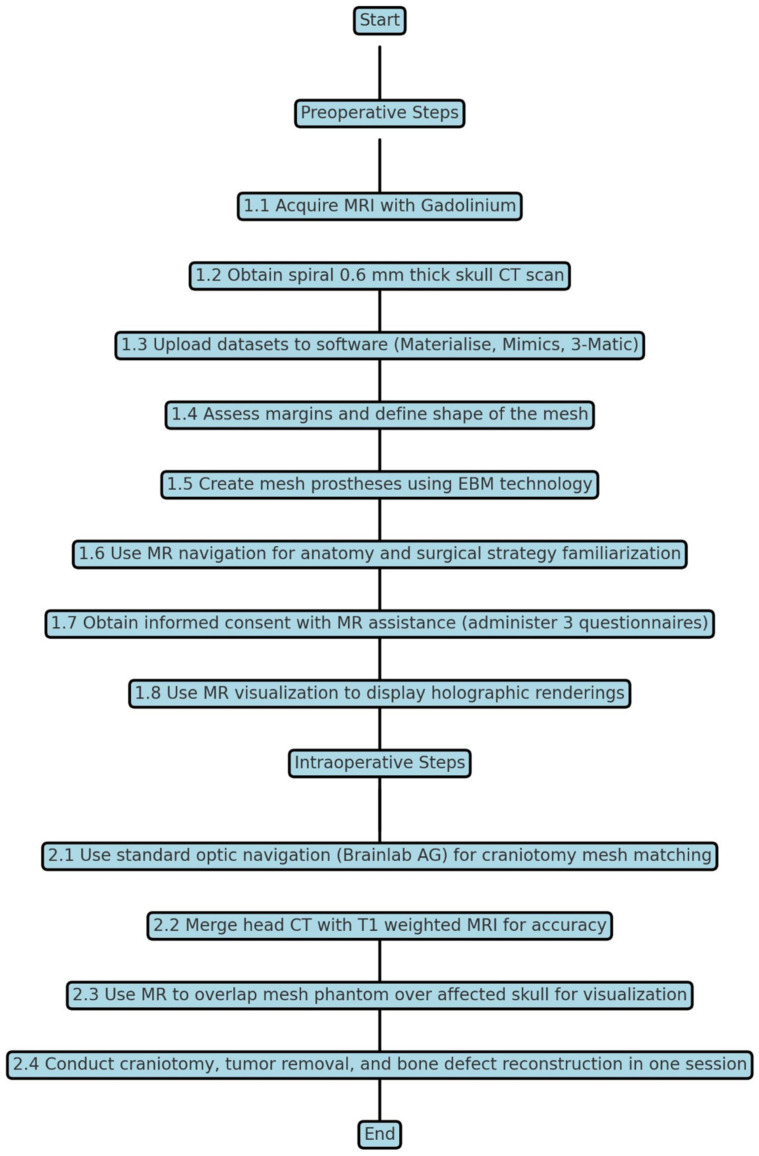
This flowchart outlines the sequence of actions taken during the preoperative and intraoperative phases, highlighting the integration of advanced imaging, modeling, and visualization techniques to enhance surgical precision and patient engagement.

We conducted a retrospective analysis of 14 consecutive patients treated for tumors invading the skull at the Department of Neurosurgery, Cannizzaro Hospital, Catania, Italy. Exclusion criteria included the following: (i) patients with high-grade malignant tumors requiring multi-staged resection; (ii) systemic comorbidities precluding general anesthesia or implant placement; (iii) prior cranial reconstruction or infection at the surgical site; and (iv) insufficient imaging data or inability to manufacture a custom prosthesis in time for a single-stage procedure. Surgical intervention aimed to remove the tumor and restore skull continuity for both cosmetic and protective purposes. A custom-made titanium alloy mesh was employed in all cases. Intraoperative standard navigation was augmented with MR navigation to enhance the precision of the resection and reconstruction procedures. The introduction of the MR head-mounted display (Hololens 2, Microsoft^TM^, Redmond, WA, USA) at our department aimed to evaluate this technology and explore its potential applications in daily neurosurgical practice. The Virtual Surgery Intelligence platform (VSI Manager, ApoQlar GmbH, Hamburg, Germany) was utilized to upload anonymized datasets, including baseline head CT scans, DICOM datasets of the phantom prosthesis, and post-cranioplasty head CT scans. These datasets were processed remotely: after uploading to the VSI cloud, 3D holographic models of the preoperative and postoperative images, as well as the holographic representation of the prosthesis, were generated. Subsequently, the hologram was downloaded from the cloud and viewed overlaid on the patient’s head using the MR headset. Tumor resection margins were meticulously planned in multiple stages (Figure 1).

### 2.1. Preoperative Steps

**Figure 2:**
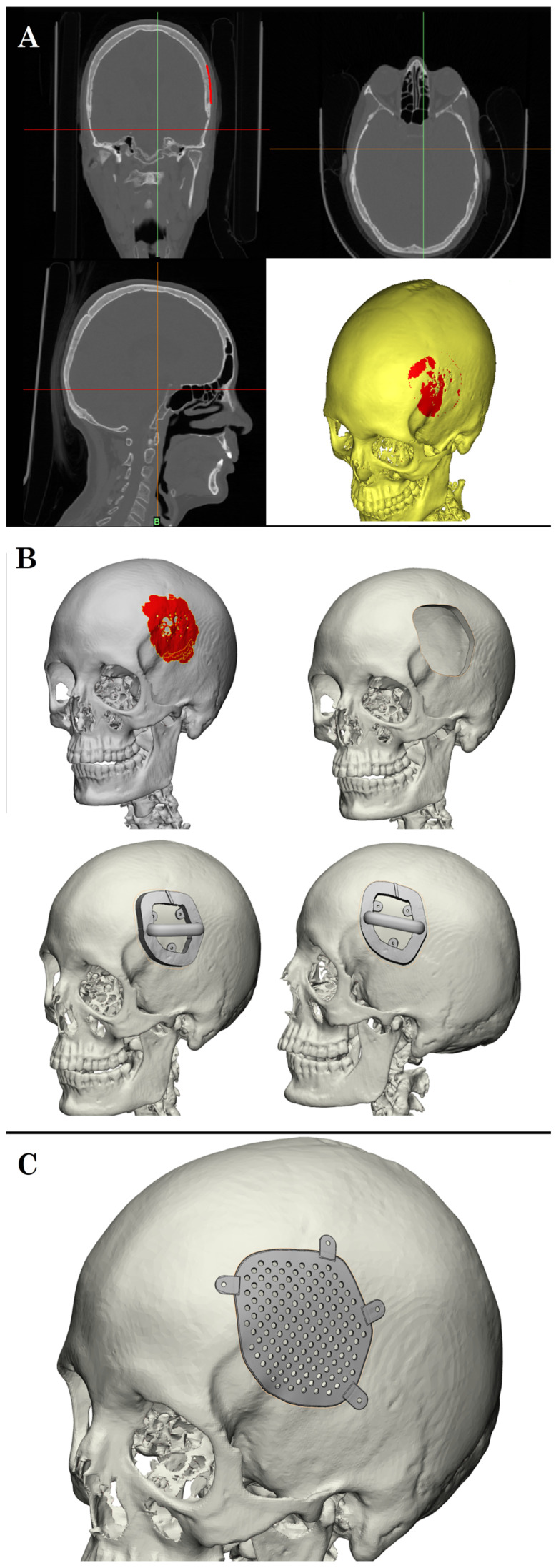
(**A**) Workflow for the preoperative planning of one-step cranioplasty begins with volumetric MRI and CT imaging acquisition. (**B**) These datasets are processed using Materialise software—Mimics for CT segmentation and 3-Matic for 3D modeling and design of the custom titanium mesh prosthesis. (**C**) The custom-made prosthesis is virtually designed to precisely fit the cranial defect, ensuring optimal tumor resection margins and enabling accurate anatomical reconstruction. MR navigation was then used to enhance preoperative familiarity with patient anatomy and facilitate surgical planning. The MR headset provided simultaneous visualization of the skull, tumor, and prosthesis in a 3D holographic environment, allowing for intuitive spatial understanding beyond what standard navigation systems offer (Figure 3).

Each patient underwent a contrast-enhanced volumetric T1-weighted MRI with gadolinium and a high-resolution Multiplanar Reformation (MPR) volumetric computed tomography (CT) scan of the skull (0.6 mm slice thickness). These datasets were processed using Materialise software—Mimics 27.1 for CT segmentation and 3-Matic for 3D modeling and design of the custom titanium mesh prosthesis. Tumor resection margins were defined through semi-automatic segmentation of the MRI, with manual refinement of enhancement thresholds and validation through radiological–surgical cross-correlation to ensure precise en bloc resection. The prostheses were manufactured using electron beam melting (EBM) technology (MT Ortho Srl, Aci Sant’Antonio, Catania, Italy) (Figure 2).

### 2.2. Intraoperative Steps

Standard optical navigation (Brainlab AG, Munich, Germany) was used to align the craniotomy with the preoperative plan and guide precise prosthesis placement. Fused CT and MRI data enhanced anatomical detail and accuracy. MR navigation enabled real-time projection of the mesh and tumor holograms directly onto the patient’s head, allowing the surgical team to maintain focus within the operative field without consulting external monitors. Craniotomy, tumor resection, and custom mesh reconstruction were performed sequentially during the same operative session.

### 2.3. Descriptive Statistical Note

Descriptive statistical analyses were performed using basic spreadsheet functions (Microsoft Excel, Redmond, WA, USA), reporting means and standard deviations. Given the small sample size and retrospective design, no formal hypothesis testing was performed.

## 3. Results

### 3.1. Patient Population

The study cohort consisted of 14 patients with a balanced sex distribution (7 male, 7 female) and a mean age of 52.6 ± 10.8 years (range: 35–74) presenting with tumors involving the diploic space: 9 meningiomas (including 1 left fronto-orbital, 1 crossing the superior sagittal sinus with bilateral extension, 3 parasagittal, and 4 supratentorial convexity) and 4 patients with other etiologies (squamous cell carcinoma, diploic hemangioma, arachnoid cyst, osteoid osteoma, and giant cell tumor of the convexity) (Table 1). Complementary treatments included adjuvant therapies such as radiotherapy or systemic pharmacological agents (e.g., denosumab) tailored to histological diagnosis and recurrence risk.

### 3.2. Titanium Custom-Made One-Step Cranioplasty

For all patients with tumors involving the intraosseous space, a volumetric preoperative CT scan was conducted to guide the creation of a titanium-alloy custom-made cranioplasty. During surgery, neuronavigation and a physical template were used to define the edges of the craniotomy, ensuring accurate alignment with the cranioplasty. The titanium mesh is 1 mm thick with anchoring wings of 0.3 mm. Precision in craniotomy size is crucial, considering the thicker and softer nature of the diseased bone, which can complicate bony cuts with a craniotome and affect resection accuracy. Utilizing a high-speed drill proved beneficial in accurately delineating tumor resection margins.

### 3.3. Mixed Reality Planning

CT DICOM files were anonymized and uploaded to cloud software (VSI Manager, ApoQlar GmbH, Hamburg, Germany) on a laptop. The cloud viewer converted the DICOM dataset into a proprietary file format to create a unified 3D holographic object rather than individual CT slices. The MR head-mounted display (Hololens2) was used to access and visualize the holographic 3D renderings directly from the cloud service. Additional CT scans of the plaster skull model and the phantom mesh model were obtained. MR holograms of the skull and cranioplasty were shown to patients and family members during the informed consent process. These holograms were used for preoperative cranioplasty planning and intraoperatively overlaid onto the patient’s head. Post-cranioplasty CT scans provided detailed reconstructions of bone and soft tissues with enhanced muscle visualization compared with standard CT imaging. The reliability of the holographic renderings of the cranioplasty was confirmed through comparison with physical models.

### 3.4. Informed Consent MR-Assisted

All patients completed three questionnaires, reporting high satisfaction, increased confidence, and engagement with the surgical strategy. Anxiety levels decreased in all but one patient, who preferred minimal technical details and relied primarily on trust in the surgeon. Nonetheless, this patient also expressed heightened engagement and reassurance with the overall surgical plan, despite initial apprehension. It is important to note, however, that the assessment tools used were custom-designed and not validated instruments. Although the responses provide useful preliminary insights into patient perception, future studies should incorporate standardized and validated questionnaires, such as the Quality of Informed Consent (QuIC) tools, to objectively evaluate the educational and emotional impact of MR-assisted communication.

### 3.5. Mixed Reality Accuracy Assessment

MR navigation accuracy was compared with optical navigation, both performed with patients in a supine position. Manual alignment of anatomical landmarks (orbits, nose, ears, forehead, mouth) with the holographic object overlaid on the patient’s skin was used. Optical navigation showed an accuracy range of 0.6–1 mm, while holographic navigation ranged from 0.2–2.8 mm. In our series, the absolute registration error for MR navigation ranged from 0.2 to 2.8 mm, with a mean error of 1.3 ± 0.7 mm (mean ± SD), while the standard optical navigation consistently maintained an error range of 0.6–1 mm. Although these descriptive results favor standard navigation for precision, MR navigation offered notable advantages in terms of real-time holographic overlays and an enhanced field of view. While no inferential statistical testing was performed due to the limited sample size, the descriptive accuracy data suggest that standard optical navigation currently offers greater precision. Nevertheless, MR provides ergonomic and educational advantages that may justify its integration into surgical workflows. Future improvements—such as fiducial-based or QR code-based registration as well as operating room lighting adjustments like blue-green illumination—may significantly reduce MR registration errors and bridge the current accuracy gap.

### 3.6. Follow-Up

Follow-up evaluations included brain MRI with gadolinium, skull CT scans, whole-body CT scans with contrast (for metastatic patients), and 68-GADOTATOC-PET scans (for meningioma patients) spanning from 6 months to 5 years. No cases of recurrence were observed, and none of the patients experienced neurological deficits. Complications included CSF leakage and wound infections in two patients, managed successfully with antibiotics, seriated medication, compression bandages, and acetazolamide therapy as needed. One patient initiated denosumab therapy with stable clinical outcomes.

## 4. Discussion

Skull tumors may present as a spontaneous, usually painless deformity with a consistent aesthetic impact. The surgical treatment aims to remove the lesion, obtain a histological diagnosis, and, if needed, facilitate complementary treatments while restoring cerebral protection and aesthetics. Several materials can be used to replace the affected bone, including methacrylate, autologous, hydroxyapatite, titanium implants, PEEK, and mesh devices [6,7].

To identify the best strategy for this complex group of patients, previous studies have proposed therapeutic algorithms considering the type of defect, material, and tumor histology [7]. Regarding materials, two main categories can be identified: handcrafted, ready-to-use options (e.g., methyl methacrylate and mesh) and custom-made prostheses (e.g., hydroxyapatite, titanium mesh, PEEK). Methyl methacrylate remains widely used due to its affordability and ease of use [8], but we avoid it because its aesthetic outcome is often unpredictable and operator-dependent. Conventional mesh systems are versatile and partially handcrafted, offering low-cost alternatives in urgent settings [6].

Hydroxyapatite prostheses are particularly suitable for young patients with traumatic cranial defects, given their osteoinductive properties. However, in oncological cases, we prefer inert materials like titanium or PEEK, which offer better handling and stability [9,10]. Custom-made models—regardless of material—are derived from CT scan DICOM data, enabling accurate preoperative planning. Surgical precision in reproducing the planned craniotomy is crucial not only for aesthetics but also for oncological control, as these tumors, although often benign, may invade eloquent structures such as venous sinuses, orbits, or paranasal regions [11].

In our series, we exclusively used custom-made titanium alloy meshes manufactured locally to reduce production times. Titanium was selected over PEEK and hydroxyapatite due to its thin profile (1 mm), favorable cosmetic outcome, and ease of intraoperative manipulation, with minimal need for bone remodeling [12]. Although PEEK may offer slightly improved resistance to infection [13], no significant infectious complications were observed in our cohort.

In the context of MR technology, recent experimental studies have explored strategies to improve spatial alignment.

Marrone et al. [14] demonstrated that blue-green light (BGL) illumination can enhance holographic neuronavigation accuracy, mitigating the glare caused by standard operating room lighting. Although not employed in our study, this approach may help optimize hologram visibility and reduce registration error, narrowing the gap between MR and conventional systems.

Given the reliance on precise alignment with custom implants, we routinely use intraoperative navigation to match the planned craniotomy with the final prosthesis. Standard optical/magnetic navigation (SN) and MR were both integrated in our workflow. Titanium mesh was selected for its balance of rigidity, flexibility, and availability. SN remains superior in precision, owing to its quantitative surface registration, while MR relies on manual anatomical alignment, which introduces variability. However, MR provides critical preoperative advantages, including immersive anatomical visualization and improved patient-specific planning. Intraoperatively, it enables “heads-up” navigation with real-time visualization of the holographic craniotomy and prosthesis directly on the patient, without requiring screen consultation.

Regarding the navigation modality, we used standard navigation in comparison with MR navigation. The rationale behind the use of MR is to introduce an innovative tool that enhances the surgeon’s understanding of the final cosmetic result [15,16]. In this procedure, reconstruction—and therefore the aesthetic outcome—is just as important as the tumor resection phase [17]. The ability to visualize MRI scans directly through the patient, rather than continuously referring to a separate monitor, significantly improves spatial awareness and facilitates a more intuitive understanding of the anticipated outcome.

Previous applications of MR in neurosurgery have primarily focused on tumor resection and vascular lesion guidance. Multiple studies have shown that MR reduces cognitive load and enhances intraoperative orientation. Our findings are consistent with these results, particularly in terms of preoperative planning. However, manual surface matching in MR continues to limit its intraoperative accuracy, as also reported in the literature. Emerging solutions—including fiducial or QR code-based registration—are being developed to address this limitation [18].

## 5. Limitations and Statistical Considerations

This study presents several limitations. The small sample size and the retrospective, single-center design limit both the statistical power and the generalizability of our findings. Selection bias may have influenced our results, as the cohort exclusively included patients with tumors infiltrating the skull and suitable for single-stage resection and reconstruction. All patients were treated with the same surgical strategy, ensuring consistency in technique. However, we excluded patients with malignant or high-grade tumors requiring multi-staged approaches, as well as those without skull invasion. This limits the generalizability of our findings, which apply primarily to benign or locally aggressive tumors that allow for en bloc resection and immediate cranioplasty. As such, our conclusions may not extend to more complex or heterogeneous neuro-oncological populations. However, due to the rarity of tumors invading the skull, and considering that this is the largest series reported to date, we believe that our findings remain of significant interest and provide valuable insights into this uncommon clinical scenario. Due to the limited cohort, we were unable to perform advanced statistical analyses such as multivariate regression or propensity score matching. Furthermore, the absence of a control group treated exclusively with standard navigation prevents direct comparison and limits conclusions regarding the added clinical value of MR. Future studies should incorporate prospective, randomized designs to provide higher-level evidence. Finally, while patient satisfaction and surgeon experience were favorable, these subjective elements are inherently difficult to measure reliably. The use of validated questionnaires and standardized outcome measures in future research will help better assess the perceived benefits of MR technology.

## 6. Future Directions and Perspectives

The demand for custom-made implants in complex craniofacial reconstruction is increasing, driven by advances in manufacturing and the growing accessibility of intraoperative visualization technologies. A multicenter prospective study or registry-based initiative would allow for the inclusion of a broader patient population, enhancing statistical power and external validity. Collaborations between high-volume centers could enable meaningful subgroup analyses (e.g., benign vs. malignant tumors, age groups), helping to identify which patients benefit most from MR-assisted navigation. Simultaneously, technical improvements such as fiducial-based, semi-automatic, or artificial intelligence (AI)-assisted hologram registration are expected to reduce MR alignment errors, potentially closing the accuracy gap with conventional optical navigation. As hardware and software evolve, MR may offer even greater advantages in real-time surgical guidance, including shorter operative times and lower complication rates. Although MR systems require dedicated hardware and cloud-based software, their increasing availability is likely to reduce upfront costs. The learning curve, while initially steep, becomes manageable after a few training sessions, with noticeable gains in efficiency. Future studies should also investigate the use of specialized lighting—such as blue-green or yellow-green illumination—to improve hologram visibility and reduce glare from overhead surgical lights. Ultimately, integrating MR into a standardized neuro-oncological workflow—from preoperative planning and patient counseling to intraoperative navigation and postoperative follow-up—has the potential to enhance surgical precision, improve patient comprehension, and support better long-term outcomes in the treatment of skull-infiltrating tumors.

## 7. Conclusions

In this study, we have presented the largest case series to date of one-step craniotomy for the removal of tumors invading the skull combined with immediate reconstruction in a single surgical session. We have also described in detail the standardized workflow adopted at our institution to manage these complex procedures. A key innovation introduced in our approach is the integration of MR-based navigation, which plays multiple essential roles: enhancing the informed consent process, improving patient engagement, facilitating preoperative anatomical understanding for the surgical team, and supporting intraoperative navigation. Looking forward, the incorporation of AI-assisted registration for hologram alignment is expected to significantly enhance the precision and reliability of MR-based navigation. This technology already enables real-time visualization of the prosthetic mesh hologram projected over the skull during surgery, while also assisting in postoperative evaluation of soft tissue reconstruction, thereby advancing both the technical execution and conceptual understanding of skull-infiltrating tumor surgery.

## Figures and Tables

**Figure 3:**
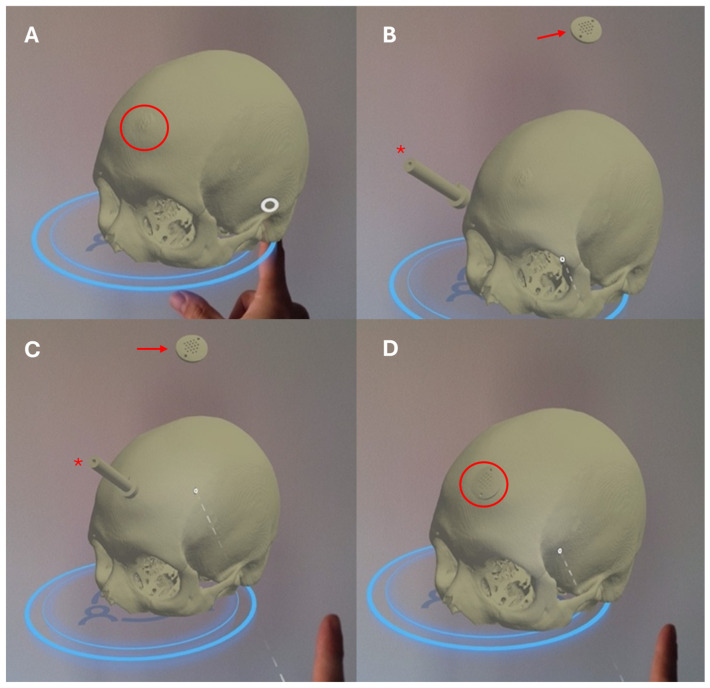
(**A**) The skull lesion is clearly visible infiltrating the frontal bone (red circle). (**B**) Multiple-object navigation is demonstrated, simultaneously displaying the skull, the infiltrated bony segment, the prosthetic template (dima) (red asterisk), and the hologram of the final implant (red arrow). (**C**) The prosthetic template (red asterisk) is placed directly over the infiltrated area of the skull, while the final implant is shown above it (red arrow). A white circle indicates a key anatomical reference point used for aligning the prosthetic elements, and the dashed line represents the planned resection margin or boundary of the affected bone. Finally, (**D**) presents the holographic simulation of the completed procedure, illustrating how the implant precisely covers the bony defect after resection (red circle).

**Table 1:** Summary of clinical characteristics and surgical outcomes for the 14 patients treated for skull-invading tumors. The table details patient demographics, tumor types (including meningiomas and other etiologies), surgical techniques, materials used for cranioplasty, and postoperative results, including any complications and follow-up information.

	Age	Sex	Tumor Location	Histology	Complementary Treatments (Radiotherapy/Chemotherapy)	Mesh Material	Size (cm^3^)	Infiltrated Eloquent Structures	Follow-Up
1	74	M	Convexity	Squamous cell carcinoma	yes	3D printed titanium mesh	58	no	1 years
2	35	F	Left frontal	Diploic hemangioma	none	3D printed titanium mesh	47	no	1 year
3	48	M	Supratentorial convexity, right frontal	Arachnoid cyst	none	3D printed titanium mesh	80	no	3 years
4	51	F	Convexity	Osteoid osteoma	none	3D printed titanium mesh	84	no	2 years
5	49	M	Right frontal parasagittal	Giant cell tumor of the convexity	Denosumab	3D printed titanium mesh	45	no	6 months
6	41	F	Left fronto-orbital	Meningioma	none	3D printed titanium mesh	53	yes	2 years
7	48	M	Vertex crossing the superior sagittal sinus with bilateral extension	Meningioma	none	3D printed titanium mesh	142	yes	3 years
8	68	M	Parasagittal	Meningioma	none	3D printed titanium mesh	60	no	6 months
9	60	F	Parasagittal	Hemangioma	none	3D printed titanium mesh	66	no	1 year
10	46	F	Spheno-orbital	Meningioma	none	3D printed titanium mesh	61	yes	1 year
11	55	M	Supratentorial convexity	Meningioma	none	3D printed titanium mesh	68	yes	3 years
12	57	M	Supratentorial convexity	Meningioma	none	3D printed titanium mesh	86	no	2 years
13	56	F	Left frontal	Meningioma	none	3D printed titanium mesh	79	no	3 years
14	45	M	Supratentorial convexity	Meningioma	none	3D printed titanium mesh	115	no	3 years

## Data Availability

The original contributions presented in this study are included in the article. Further inquiries can be directed to the corresponding author.
